# Bioinformatic analysis of eosinophil activity and its implications for model and target species

**DOI:** 10.1017/S0031182019001768

**Published:** 2020-04

**Authors:** C.J. Jenvey, D. Alenizi, F. Almasi, C. Cairns, A. Holmes, S. Sloan, M.J. Stear

**Affiliations:** Department of Animal, Plant and Soil Sciences, Agribio Centre for Agribioscience, School of Life Sciences, La Trobe University, Bundoora, Victoria, Australia

**Keywords:** Bioinformatics, CCR3, eosinophils, EPX, Fc*α*RI, gastrointestinal nematodes, PRG3, ruminants

## Abstract

Eosinophils are important immune cells that have been implicated in resistance to gastrointestinal nematode (GIN) infections in both naturally and experimentally infected sheep. Proteins of particular importance appear to be IgA-Fc alpha receptor (Fc*α*RI), C-C chemokine receptor type 3 (CCR3), proteoglycan 3 (PRG3, major basic protein 2) and EPX (eosinophil peroxidase). We used known human nucleotide sequences to search the ruminant genomes, followed by translation to protein and sequence alignments to visualize differences between sequences and species. Where a sequence was retrieved for cow, but not for sheep and goat, this was used additionally as a reference sequence. In this review, we show that eosinophil function varies among host species. Consequently, investigations into the mechanisms of ruminant immune responses to GIN should be conducted using the natural host. Specifically, we address differences in protein sequence and structure for eosinophil proteins.

## Introduction

Host immune responses to gastrointestinal nematodes (GINs) are dominated by a Th2 immune response; involving antibodies and immune cells, such as immunoglobulin A (IgA), IgE, mast cells and eosinophils. In particular, ruminants naturally and experimentally infected with GIN demonstrate an increase in blood and tissue eosinophilia, implying that eosinophils may be an important mediator of host immune responses to GIN. However, both phenotypic and bioinformatic evidence suggest that eosinophil activity against GIN may differ between hosts (Urban *et al*., 1991; Henderson and Stear, 2006). Bioinformatic analyses on eosinophil-associated proteins were used to explore whether differences in resistance to GIN among species were genetic in origin. Specifically, we addressed differences in protein sequence and structure for eosinophil proteins. These proteins included IgA and its receptor, Fc*α*RI, interleukin (IL)-5 and its receptor, IL-5R*α*, eotaxin and its receptor, CCR3, major basic protein (MBP, PRG3) and eosinophil peroxidase (EPX). We used known human nucleotide sequences to search the ruminant genomes (*Bos taurus*, cow; *Ovis aries*, sheep; *Capra hircus*, goat), retrieved sequences (Supplementary Table 1), followed by translation to protein and sequence alignments to visualize differences between sequences and species.

### Eosinophils and GIN infections

Eosinophils are a sub-type of granulocyte, along with mast cells, neutrophils and basophils. Following proliferation of eosinophil precursors from the bone marrow, eosinophils traffic to sites of infection and are activated. Once activated, eosinophils undergo degranulation, releasing cytotoxic proteins from secondary granules to protect the host against foreign pathogens. Eosinophils are also involved in immune homoeostasis and immunity (Rothenberg and Hogan, 2006; Weller and Spencer, 2017). Eosinophils are found in both blood and tissue, however the gastrointestinal tract contains the largest reservoir of eosinophils in the body (Zuo and Rothenberg, 2007) and only tissue eosinophils degranulate (Blanchard and Rothenberg, 2009). The ability of eosinophils to defend the host against parasitic helminths is suggested by the ability of eosinophils to mediate antibody- (or complement-) dependent cellular cytotoxicity (ADCC) *in vitro* and *in vivo* (Giacomin *et al*., 2008; Huang *et al*., 2015), increased numbers of eosinophils during helminth infections, as well as degranulation in close proximity to helminths *in vivo* (Rothenberg and Hogan, 2006).

Eosinophils are not only important for GIN infections of humans and mice, but also of ruminants. In particular, GIN infections ravage sheep and goat populations in temperate regions of the world, and in Australia can cost sheep producers up to $500 million per year largely in lost productivity (Lane *et al*., 2015). In particular, *Teladorsagia circumcincta*, *Haemonchus contortus* and *Trichostrongylus colubriformis* are the dominant GIN-infecting small ruminants (Roeber *et al*., 2013). The typical immune response to GIN is dominated by Th2 immune responses, namely the production of antibodies such as IgG, IgE and IgA, as well as involvement of mast cells and eosinophils, the details of which have previously been covered by a number of reviews (Maizels and Yazdanbakhsh, 2003; Anthony *et al*., 2007; McRae *et al*., 2015; Motran *et al*., 2018). Previous research using experimentally and naturally infected sheep have indicated that eosinophils may play an important role in resistance to infection. Investigations into sheep immune responses to *T. circumcincta* (Gruner *et al*., 1994; Stear *et al*., 1995, 2002; Henderson and Stear, 2006; Beraldi *et al*., 2008), *H. contortus* (Rainbird *et al*., 1998; Gill *et al*., 2000; Balic *et al*., 2006; Terefe *et al*., 2007, 2009) and *T. colubriformis* (Dawkins *et al*., 1989; Rothwell *et al*., 1993; Amarante *et al*., 2007) have all demonstrated increases in eosinophils in resistant animals, resistant breeds and/or in sheep selectively bred for resistance. In addition, differences in numbers of eosinophils and susceptibility to infection have also been observed in goats (Bambou *et al*., 2013; Basripuzi *et al*., 2018).

Such findings imply that eosinophils are important cells in ruminant responses to GIN infections. Phenotypic and bioinformatic evidence suggests that there are differences in immune responses to GIN between species. A recent review by Weller and Spencer (2017) discussed a number of unresolved issues when comparing mouse and human eosinophils, namely whether the formation and secretion of eosinophil cytokines is regulated by common mechanisms. In addition, a review by Behm and Ovington (2000) highlighted that IL-5 and eosinophils have different impacts on different helminth infections. Conversely, a review by Meeusen and Balic (2000) suggests that the presence of IL-5 independent eosinophil populations within tissue and peripheral blood may play a role in unnatural nematode-mouse models by increasing resistance to primary infections, and enhancing the development of specific immunity upon subsequent infections. Ultimately, although a number of *in vitro* studies investigating the mechanisms by which eosinophils cause helminth death have been demonstrated, it is not yet clear whether these same mechanisms also occur *in vivo* (Motran *et al*., 2018). Recent updates to the human, mouse, cow, sheep and goat genomes have provided insights into the possible mechanisms of eosinophil function, which can be used to direct functional studies into the mechanisms of eosinophils during GIN infections of ruminants.

## The IgA receptor, Fc*α*RI, may be dysfunctional in goats

Immunoglobulin A (IgA) is an antibody that plays a crucial role in the immune function of mucus membranes. TGF-*β*, together with IL-5, is responsible for class-switching of B lymphocytes into IgA-producing plasma cells (Coffman *et al*., 1989; Sonoda *et al*., 1989). This local production of IgA is termed secretory IgA and is the predominant form of IgA in mucosal secretions (van Egmond *et al*., 2001; Bakema and van Egmond, 2011). Considering the importance of IgA in the protection of mucus membranes, it is unsurprising that studies investigating immune responses to parasitic infections have found associations between IgA and parasite-induced eosinophilia (Muraki *et al*., 2011). IgA has been demonstrated to be associated with nematode fecundity and peripheral eosinophils, and therefore resistance to helminth infection in sheep (Gill *et al*., 1993; Henderson and Stear, 2006; Halliday *et al*., 2007; Hernández *et al*., 2016; Fairlie-Clarke *et al*., 2019). The lack of an effective IgA response has also been implicated in increased susceptibility to parasite infection in goats (Basripuzi *et al*., 2018). Additionally, *in vitro* studies have indicated that parasite-induced eosinophil cytolysis may be dependent upon IgA binding to its receptor (Ueki *et al*., 2013).

IgA exists as a monomer of two identical heavy chains and two identical light chains, but can form a dimer (secretory IgA) stabilized by disulphide bonds and a joining (J) chain (Woof and Russell, 2011). In ruminants, most IgA in serum derives from mucosal surfaces and is largely dimeric (Scicchitano *et al*., 1986). The Fc*α*RI (CD89) is a transmembrane receptor and due to heavy glycosylation, eosinophil Fc*α*RI is heavier (70–100 kDa) compared to macrophage and neutrophil Fc*α*RI (55–75 kDa) (van Egmond *et al*., 2001). Fc*α*RI comprises a ligand-binding alpha chain, which consists of two extracellular Ig-like domains, a transmembrane domain, and a short cytoplasmic tail. Due to the lack of signalling motifs, Fc*α*RI must associate with the FcR*γ* chain for signalling and function (van Egmond *et al*., 2001).

**Fig. 1.**
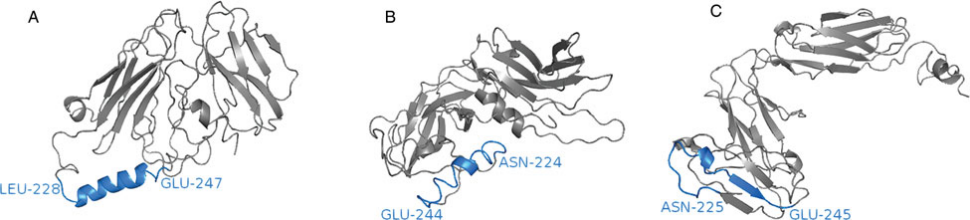
The goat IgA receptor, Fc*α*RI, may be dysfunctional in goats. Homology modelling of human, P24071.1 (A); sheep, XM_027976143.1 (B) and goat, XM_018059931.1 (C) IgA receptors revealed that human and sheep receptors have a similar conformation, however, the goat receptor has a C-terminus that is bent away from the main body of the protein and lacked an alpha helix within the transmembrane domain (blue).

Molecular modelling of the eosinophil IgA receptor indicates that it may be dysfunctional in goats (Basripuzi *et al*., 2018). Structural modelling of the IgA receptor indicated that the sheep and human receptors had similar structures. However, the goat receptor had a different conformation, with the C-terminus being bent away from the main body of the protein and lacked an alpha helix within the transmembrane domain (approximately 20 amino acids) (Fig. 1). Although the sheep and goat sequences showed high sequence identity (96%), the sheep and goat sequences showed only 55 and 54% sequence identity with the human protein. Additionally, a difference of only three amino acids was observed within the binding domain (EC1) and no differences were observed within the transmembrane domain. This seems to indicate that the loss of secondary structure within the transmembrane domain is most likely a consequence of sequence differences outside of this domain. As yet, no mouse homologue of Fc*α*RI has been identified (van Egmond *et al*., 2001; Decot *et al*., 2005), thus if eosinophils and IgA interact to control nematodes through ADCC, mice may not be appropriate models for studying eosinophil activity against nematodes infecting humans and ruminants.

## Differences in eosinophil responsiveness are not due to IL-5 and IL-5R*α*

IL-5 is a growth factor and chemoattractant of eosinophils and is involved in the recruitment, activation, degranulation and survival of eosinophils (Lopez *et al*., 1988; Horie *et al*., 1996; Fulkerson *et al*., 2014; Sippel *et al*., 2018). IL-5 is an important cytokine in the differentiation and activation of anti-parasitic eosinophil responses and has been specifically targeted in mice to demonstrate the *in vivo* role of eosinophils in helminth infections, as discussed in previous reviews (Huang and Appleton, 2016; Meeusen and Balic, 2000). However, the reagents and knock-out models used in these rodent studies are generally not available or suitable for large animal experimentation. Several studies have shown that mice vaccinated with recombinant cytokines can induce autoantibodies that specifically cancel out the activity of the native cytokine *in vivo* (Dalum *et al*., 1999; Hertz, *et al*., 2001; Richard *et al*., 2000). This approach may provide an alternative for the *in vivo* study of immune responses in large animals.

IL-5 acts on target cells by binding to its receptor, IL-5R, which consists of an *α* and a *β* subunit, however the *α* subunit is specific to IL-5R only (McBrien and Menzies-Gow, 2017). IL-5 is a dimeric glycoprotein with a four-helix bundle motif. In complex, IL-5 forms a homodimer which is sandwiched by the IL-5R*α*. Binding of IL-5 to the receptor alpha subunit results in recruitment of the *β* subunit to the receptor (Tavernier *et al*., 1991; Kusano *et al*., 2012). The human and mouse cDNA code for proteins of 134 and 133 amino acids in length, respectively, and have 70% amino acid sequence identity (Yamaguchi, 1994).

Molecular modelling indicates that it is unlikely that IL-5 and its receptor are responsible for differences in eosinophil responses between sheep and goats. For IL-5, the predicted protein sequences were identical between sheep and goat, while for IL-5R*α*, the predicted protein sequences differed in 6 out of the 432 amino acids. All of these differences occurred in the first 42 amino acids of the protein, which included within the 15–34 amino acids that aligned with the signal peptide for human IL-5R*α*, as well as within a region lacking secondary structure. This indicates that the observed sequence differences are unlikely to result in a dysfunctional IL-5 and IL-5R.

## The eotaxin receptor, CCR3, contains a frameshift mutation in goats

Eotaxin is a chemoattractant cytokine which is important for promoting eosinophil recruitment and degranulation (Garcia-Zepeda *et al*., 1996; Davoine and Lacy, 2014), and has been shown to be important for eosinophil recruitment during helminth infections (Rothenberg *et al*., 1997; Mochizuki *et al*., 1998; Ruth *et al*., 1998; Culley *et al*., 2000; Simons *et al*., 2005). Eotaxin belongs to the CC chemokine family, which is distinguished by two cysteines immediately adjacent to the N terminus. Eotaxin has been determined to be in equilibrium between a monomer and a dimer at near physiological pH, however, functional eotaxin is present as a monomer (Crump *et al*., 1998). It exhibits a chemokine-like fold consisting of three anti-parallel *β*-strands with an overlying *α*-helix (Crump *et al*., 1998). There are three molecules of Eotaxin, CCL11 (Eotaxin-1), CCL24 (Eotaxin-2) and CCL26 (Eotaxin-3); however, Eotaxin-1 is the dominant isoform. The highest levels of Eotaxin-1 are found in the GI system and can be produced by a variety of cells (Kitaura *et al*., 1996; Ying *et al*., 1999). Eotaxin-1 is important for the release of eosinophil precursors from the bone marrow (Palframan *et al*., 1998) and is activated by Th2 cytokines (Mochizuki *et al*., 1998) and inhibited by Th1 cytokines (Miyamasu *et al*., 1999; Fukuda *et al*., 2002). Eotaxin-2 is synthesized and released by mucosal epithelial cells and macrophages, while Eotaxin-3 is produced by epithelial and endothelial cells (Kitaura *et al*., 1999; Shinkai *et al*., 1999; Dulkys *et al*., 2001). Eotaxin-2 and Eotaxin-3 can also recruit eosinophils, but at later stages of infection (>24 h) (Ying *et al*., 1999; Rosenwasser *et al*., 2003; Kalomenidis *et al*., 2005; Schratl *et al*., 2006).

All Eotaxin isoforms are associated with a single receptor, CCR3, however the receptor binds to the different isoforms with different affinities (Kitaura *et al*., 1999). CCR3 is very abundant in eosinophils (approximately 40–400 × 10^3^ receptors per cell), but is also expressed at lower levels in basophils, mast cells and a subset of Th2 lymphocytes (Sallusto *et al*., 1997; Uguccioni *et al*., 1997; Romagnani *et al*., 1999). CCR3 also binds to other non-eosinophil selective CC chemokines, but with lower affinity compared to Eotaxin-1 (Ponath *et al*., 1996; Baggiolini *et al*., 1997; Sabroe *et al*., 1999). CCR3 is a G-protein-coupled receptor of 335 amino acids in length and shares 63 and 51% sequence homology with CCR1 and CCR2, respectively (Daugherty *et al*., 1996). The CCR3 gene codes for four cysteine residues, one in each of the extracellular domains, and a serine/threonine-rich cytoplasmic tail, all of which are highly conserved features of chemokine receptors (Ponath *et al*., 1996). Uniquely, CCR3 contains a cluster of negatively charged amino acids distal to the transmembrane helix IV in the second extracellular loop (Daugherty *et al*., 1996).

**Fig. 2.**
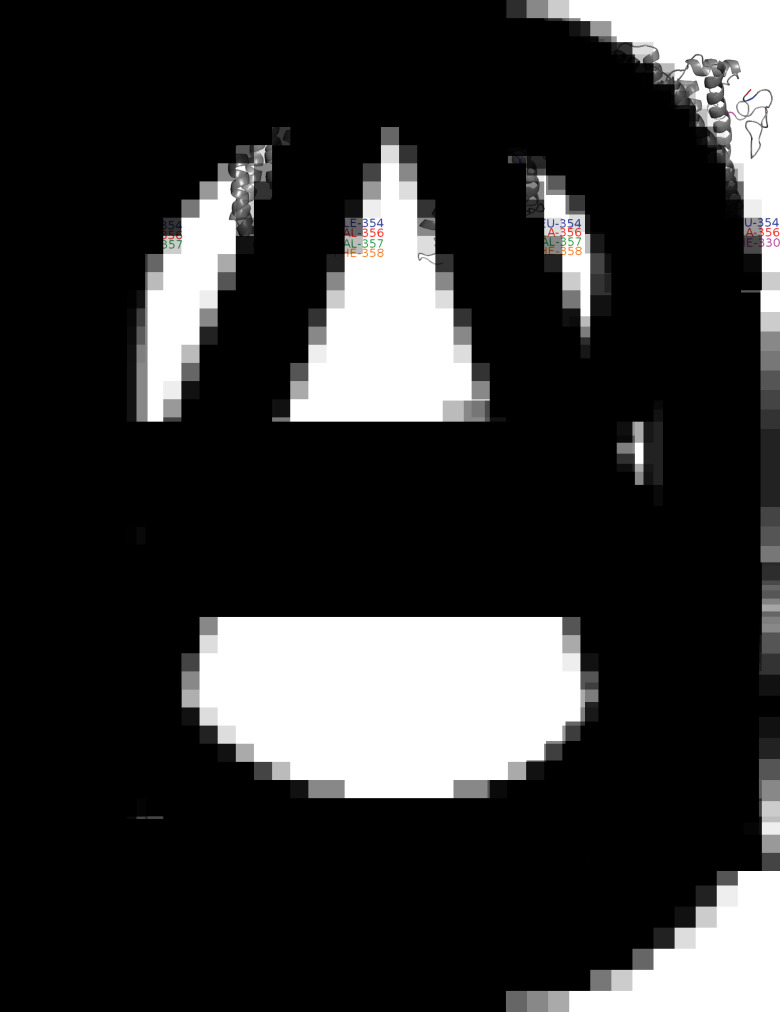
Eotaxin receptor, CCR3, may be dysfunctional in goats. Homology modelling revealed that the two sheep sequences [Q9N0M0 (B), W5PXW1 (C)] were identical except for two substitutions and all substitutions were at the C-terminal end of the sequence. The two goat sequences [JO419941.2 (A), LOC5316646 (D)] contained two substitutions compared to the sheep sequences. In addition, the goat genome sequence contained a frameshift deletion, corresponding to amino acid 330 (pink). Substitutions are colour coded by the following: aa 354 (blue), aa 356 (red), aa 357 (green) and aa 358 (orange).

Molecular modelling of the Eotaxin receptor, CCR3, indicates that it may be dysfunctional in goats. The sheep and goat CCR3 protein sequences differed by only two amino acids. The two sheep protein sequences were identical except for two substitutions. The two goat sequences were derived from the goat genome sequence and the other from mRNA extracted from the liver of an Osmanabadi goat. The goat genome sequence contained a frameshift deletion on chromosome 22 at 52,155,650, which corresponded to amino acid 330 of the CCR3 protein (Fig. 2). This deletion was not present in the mRNA; therefore, it is possible that this deletion is merely a sequencing artefact. Additionally, both goat sequences contain two amino acid substitutions in relation to the sheep sequences. However, it is unlikely that these substitutions would be the cause of any dysfunction, as the same amino acids are present in the same positions in the human CCR3 protein sequence. In any case, more research is necessary to establish if the potential frameshift mutation is polymorphic in goats and whether or not it may contribute to differences in eosinophil responsiveness between relatively resistant and susceptible goat breeds.

## MBP-2 is the only MBP molecule present in ruminants

MBP-1 is an abundant granule protein of human eosinophils. Its homologue, MBP-2, is unique to eosinophils (Acharya and Ackerman, 2014). MBP is localized within the crystalline core of the eosinophil and is an important mediator of eosinophil function (Gleich and Adolphson, 1986; Kita, 2011). MBP is highly basic, which results in the binding of MBP to cell membranes. Cytotoxic mechanisms of MBP involve surface interchange to increase cell membrane permeability and interrupting tissue enzyme activity (Ackerman *et al*., 1985; Gleich and Adolphson, 1986; Swaminathan *et al*., 2001). MBP has been demonstrated to be toxic against *Schistosoma mansoni* by disrupting the cell membrane *via* the binding of heparin (Butterworth *et al*., 1979), as well as being important in the control of *Litomosoides sigmodontis* in mice (Specht *et al*., 2006). Structurally, MBP is most like C-type lectins, except that it lacks a calcium binding site, and instead binds selectively to heparin and heparin sulphate, glycosaminoglycans and chondroitin sulphate B (Swaminathan *et al*., 2005; Wagner *et al*., 2007). Human MBP-1 is 222 amino acids long, consisting of a signalling peptide, pro-peptide and two chains (Swaminathan *et al*., 2001). It has been suggested that the pro-peptide protects the eosinophil from MBP during transport from the Golgi apparatus to the crystalline core by masking the mature domain, as well as by blocking glycosylated binding sites to inactivate the protein (Swaminathan *et al*., 2001). The mature domain is highly basic and is the region where carbohydrate recognition occurs. There is a 66% amino acid sequence identity between MBP-1 and MBP-2, with MBP-2 being less basic. MBP-2 contains 10 cysteine residues, eight of which are conserved in MBP-1, including those cysteines that are involved in disulphide bridges. The conservation of these disulphide bridges is consistent with other C-type lectins and is thought to be important for tertiary structure and function (Wagner *et al*., 2007).

**Fig. 3.**
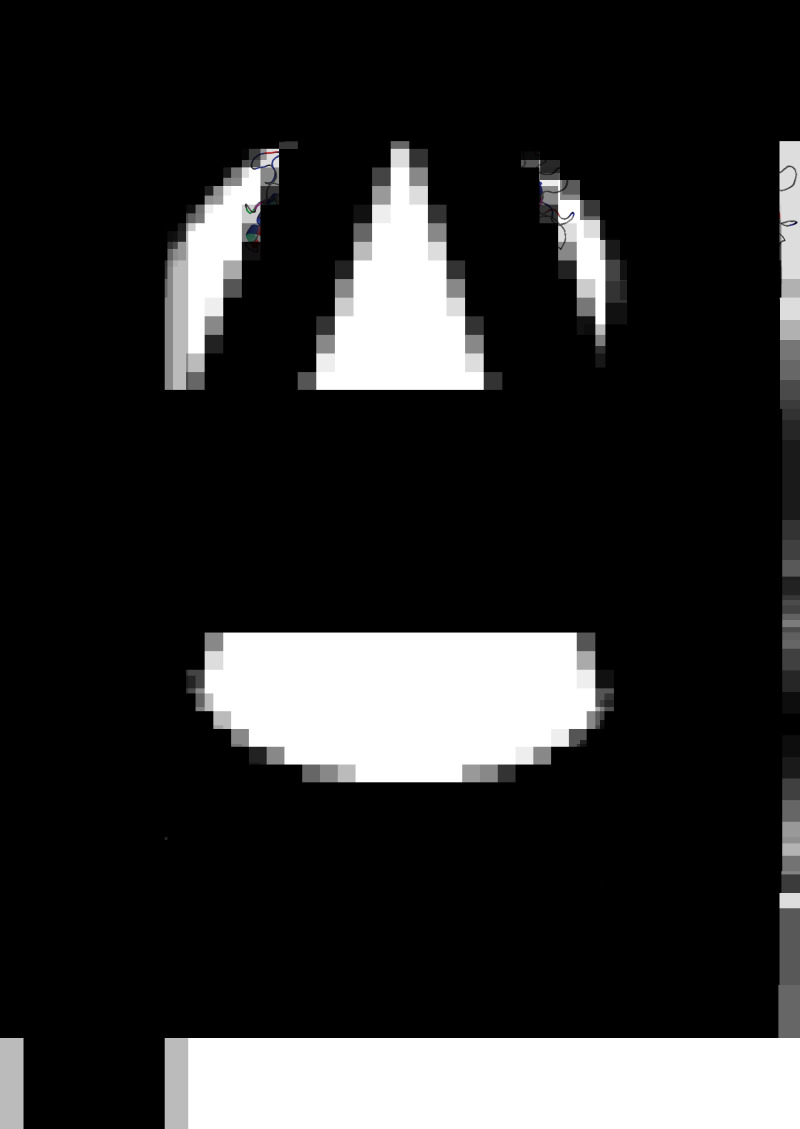
Major basic protein 2 is the only MBP molecule in ruminants. Homology modelling of MBP-1 and MBP-2 C-type lectin fold revealed high structural similarities between human MBP-2 and ruminant MBP-2 molecules, however, theoretical isoelectric point (pI) computation suggests that ruminant MBP-2 molecules may function similarly to human MBP-1. Amino acids used to calculate theoretical pI are as follows: aspartic acid (green), glutamic acid (orange), histidine (yellow), cysteine (red), tyrosine (pink), lysine (aqua) and arginine (blue). Molecules are presented in the order of percentage of sequence identity to human MBP-1, CR450311.1 (A) (highest to lowest): human MBP-2, NM_006093.4 (B); sheep XM_027979083.1 (C); sheep XM_027979084.1 (D); goat XM_018058941.1 (E); cow NM_001098471.1 (F); sheep XM_027979081.1 (G) and goat XM_018058942 (H).

Sequence searches of MBP indicate that only MBP-2 is detectable in ruminant genomes; it may function similarly to MBP-1. A total of three sequences for sheep (XM_027979083.1, XM_027979084.1 and XM_02797908.1), two sequences for goats (XM_018058941.1 and XM_018058942) and one sequence for cows (NM_001098471.1) were retrieved. Of the two human MBP sequences, the ruminant sequences were most similar to MBP-2 (55–58% homology). Homology between the sheep sequences was between 72 and 79%, while homology between the goat sequences was 79%. The highest homology was observed between sheep sequence XM_027979084.1 and goat sequence XM_018058941.1, with 95%, which implies a recent divergence and that sheep and goats may have multiple MBP loci. Typical features of C-type lectins and MBP were conserved in the ruminant sequences, including the C-type lectin fold, disulphide-bonded cysteines, and the heparin binding sites. Despite higher homology between human MBP-2 and the ruminant sequences (Fig. 3), theoretical isoelectric point (pI) calculations for the ruminant sequences were most similar to the pI for human MBP-1, with the pI of all ruminant sequences being within 26% of the pI human MBP-1, compared to within 61% of the pI of MBP-2 (Table 1). It is possible that in the absence of MBP-1, ruminant MBP-2 may function as MBP-1, being more cytotoxic and abundant. Functional assays are required to determine if this hypothesis is in fact the case.

**Table 1.** Theoretical isoelectric point (pI) of sequences for MBP molecules from humans, cows, sheep and goats

MBP sequence	Theoretical pI
Human CR450311.1	6.23
Human NM_006093.4	4.69
Cow NM_001098471.1	8.00
Sheep XM_02797908.1	6.42
Sheep XM_027979083.1	6.22
Sheep XM_027979084.1	7.96
Goat XM_018058942	6.43
Goat XM_018058941.1	8.59

Amino acid sequences were entered into the ExPASy Bioinformatics Resource Portal Compute pI/MW Tool (https://web.expasy.org/compute_pi/) for estimating average theoretical pI based upon the pK values of amino acids described in Bjellqvist *et al*. (1993) and Bjellqvist *et al*. (1994). The pK values in these studies were defined by examining polypeptide migration between pH 4.5 and 7.3; therefore, predictions for proteins outside of this pH range (cow NM_001098471.1, sheep XM_027979084.1, goat XM_018058941.1) may not be accurate.

## Goat EPX lacks a nitrosylated tyrosine

Similar to MBP-2, eosinophil peroxidase (EPX) is unique to eosinophils and is the most abundant cationic protein within the matrix of the specific granule of the eosinophil (Acharya and Ackerman, 2014). Human EPX is 715 AA long, located on chromosome 17. It is structurally similar to myeloperoxidase (MPO), which is present in neutrophil-specific granules (Loughran *et al*., 2008). EPX uses hydrogen peroxide to produce toxic reactive oxygen species, such as hypohalous acids, and is capable of killing parasites, including *S. mansoni* (Auriault *et al*., 1982), *Toxoplasma gondii* (Locksley *et al*., 1982) and *L. sigmodontis* (Specht *et al*., 2006). In addition to eosinophils, mast cells also play a role in parasitic infections. High concentrations of EPX have been demonstrated to result in mast cell lysis. Mast cell lysis is followed by the binding of EPX to mast cell granules to form a complex, which results in the retention of secretory activity on mast cells (Henderson *et al*., 1980). In addition, a study by Metzler *et al*. (2011) demonstrated donors that were deficient in MPO failed to form neutrophil extracellular traps (NET), indicating that MPO is essential for NET formation. Based on this evidence, it is possible that EPX could be involved in eosinophil extracellular trap (EET) formation, which would implicate eosinophils in the direct control of parasitic infections.

EPX is structurally distinct from the other granule proteins, being a two-chain (55-kDa heavy chain and 12.5-kDa light chain) haemoprotein, although EPX is highly cationic, much like MBP-1 and eosinophil cationic protein. A feature of EPX is that it post-translationally modifies itself *via* the nitrosylation of a specific tyrosine residue (Tyr-488) during synthesis and packaging of the granule proteins into the developing eosinophil (Ulrich *et al*., 2008). Tyr-488 has also been shown to be surface exposed, which may assist in the production of reactive oxygen species by EPX. Other granule proteins are also nitrosylated by EPX, but it is unclear whether this is important in protection of the host against helminths.

**Fig. 4.**
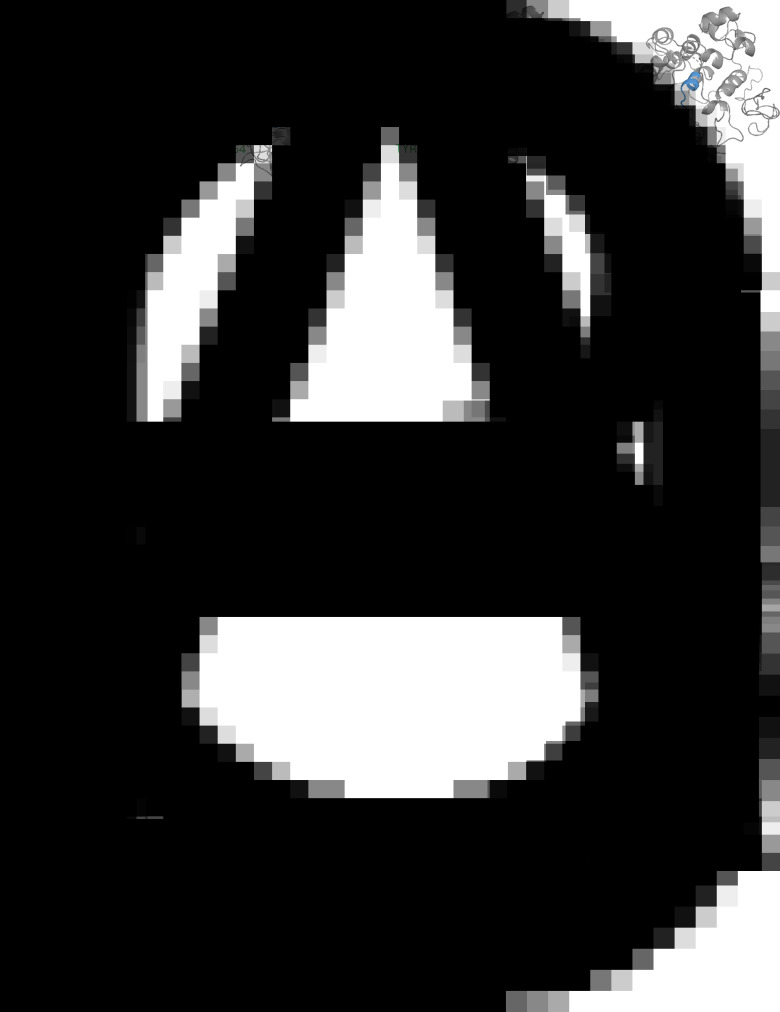
Eosinophil peroxidase may be dysfunctional in goats. Homology modelling EPX heavy chain revealed goat EPX does not contain a tyrosine involved which is involved in post-translational modification of eosinophil granule proteins during eosinophil maturation in human eosinophils. The nitrated tyrosine (green) in the human, NM_000502.6 (A); cow, XM_024980582.1 (B) and sheep, contig. AMGL01017333.1 (C) sequences have been replaced by a cysteine (red) in the goat, contig. LWLT01000022.1 (D) sequence.

Molecular modelling of EPX indicates that this protein may be dysfunctional in goats. Based on searches using the human (NM_000502.6) and bovine (XM_024980582.1) EPX sequences, no annotated sequences were retrieved from either the sheep or goat genomes. However, short sequences that matched the reference, but not annotated with an associated gene, were retrieved and re-aligned to the reference. The sheep sequence contained one single-nucleotide polymorphism (SNP), while the goat sequence contained two SNP, one of which was in the same location as in the sheep sequence. The sheep and goat sequences were 90.3% homologous, with homology of sheep and goat to the cow sequence being 84.9 and 82.9%, respectively. The cow, sheep and goat sequences all contained MPO-like protein domains, all of which were conserved except for single-residue substitutions within each domain. In addition to the MPO-like domains, the cow and sheep sequences also contained a tryptic peptide fragment, which was conserved in all ruminant sequences except for a single substitution (alignment to human sequence; Arg^483^ to His^483^). Of note, the nitrosylated tyrosine was not conserved in the goat sequence and was substituted for a cysteine (Fig. 4). The nitrosylated tyrosine is important for EPX-mediated activities, including the post-translational nitration of eosinophil secondary granule proteins, which in turn, may influence inflammatory responses. The importance of a nitrosylated tyrosine in GIN infections of ruminants has not yet been established; however, the absence of this residue may be responsible for the relative susceptibility of goats to GIN infection. Functional studies are required to determine whether the SNP identified in the sheep and goat sequences may be sequencing error rather than true SNP, as well as to confirm the importance of the nitrosylated tyrosine in resistance and susceptibility to GIN infection.

## Directions for future research

Phenotypic and bioinformatic analyses, in combination, are valuable in assessing differences in immune responses between species in order to better direct the design of functional studies. Some eosinophil proteins may be at least partly responsible for the susceptibility of certain ruminant species to GINs. In particular, sequence variation in the IgA receptor, Fc*α*RI, the Eotaxin receptor, CCR3 and EPX may contribute to the susceptibility of goats to GIN infection. The different conformation of goat Fc*α*RI, as compared to sheep and human, indicates that it is unlikely that it would be fully effective in antigen-dependent cellular cytotoxicity processes, which have been shown to be important for some helminth infections (Huang *et al*., 2015). In addition, goat CCR3 may contain a frameshift mutation, which would in turn affect the role of Eotaxin-1 in eosinophil recruitment and degranulation. Goat EPX lacks a nitrosylated tyrosine. In humans, the nitrosylated tyrosine in EPX is important for the post-translational modification of EDGPs during eosinophil maturation. Goat EPX may not be involved in this process. Finally, although MBP-1 is the most abundant and important cationic protein in human eosinophils, it has no orthologue in ruminant genomes. MBP-2 appears to be the only MBP molecule present in ruminants and it may function similarly to human MBP-1. Future research should focus on functional studies to confirm these findings and should involve the extraction of these proteins from the host of interest, followed by sequencing, crystallography and *in vitro* assays to assess the overall importance of these proteins to the susceptibility of ruminants to GIN.

Perhaps the clearest finding of this review is that bioinformatic analyses indicate that the function of specific eosinophil proteins may vary among host species. Therefore, functional studies of eosinophil activity should be performed in the target species. Extrapolations from one species to another need to be interpreted with great care.

## Supplementary material

For supplementary material accompanying this paper visit https://doi.org/10.1017/S0031182019001768. click here to view supplementary material
