# Supplementary material for: Bioinformatic analysis of eosinophil activity and its implications for model and target species
Source: Parasitology. 2019 Dec 16;147(4):393–400. doi: 10.1017/S0031182019001768 (PMC7119366; doi:10.1017/S0031182019001768)
Supplement: Supplementary file 1 [file S0031182019001768sup001.docx]

**Supplementary Table 1. Details of database searches for eosinophil proteins IgA, FcαRI, IL-5, IL-5Rα, CCR3, Eotaxin, MBP and EPX.** Abbreviations: Bt, *Bos taurus*; Ch, *Capra hircus*; Hs, *Homo sapiens*; Oa, *Ovis aries*; Mm, *Mus musculus*; ENA, European Nucleotide Archive; GB, GenBank; NCBI, National Center for Biotechnology Information; UP, UniProtKB; UP/SP, UniProtKB/Swiss-Prot.

| Protein name | Search terms | Database | Species | Accession number | Sequence details |
| --- | --- | --- | --- | --- | --- |
|  |  |  |  |  |  |
| IgA | IgA  Immunoglobulin A | GB | Ch | AMP34155.1 | Immunoglobulin alpha heavy chain, partial |
|  |  | UP/SP | Hs | P01876.2 | Immunoglobulin heavy constant alpha 1 |
|  |  | UP/SP | Hs | P01877.4 | Immunoglobulin heavy constant alpha 2 |
|  |  | GB | Mm | AAB59662.1 | Immunoglobulin alpha-chain, partial |
|  |  | GB | Oa | AAC64980.1 | Immunoglobulin alpha heavy chain, partial |
|  |  |  |  |  |  |
| FcαRI | IgA receptor  IgA Fc receptor | NCBI | Ch | XP_005693013.2 | Immunoglobulin alpha Fc receptor, predicted |
|  |  | NCBI | Ch | XM_018059931.1 | Fc fragment of IgA and IgM receptor (FCAMR), mRNA, predicted |
|  |  | NCBI | Hs | NP_001991.1 | Immunoglobulin alpha Fc receptor isoform a precursor |
|  |  | NCBI | Hs | NP_579803.1 | Immunoglobulin alpha Fc receptor isoform b precursor |
|  |  | NCBI | Hs | NP_579805.1 | Immunoglobulin alpha Fc receptor isoform c precursor |
|  |  | NCBI | Hs | NP_579806.1 | Immunoglobulin alpha Fc receptor isoform d |
|  |  | NCBI | Hs | NP_579807.1 | Immunoglobulin alpha Fc receptor isoform e |
|  |  | NCBI | Hs | NP_579808.1 | Immunoglobulin alpha Fc receptor isoform f |
|  |  | NCBI | Hs | NP_579811.1 | Immunoglobulin alpha Fc receptor isoform g |
|  |  | NCBI | Hs | NP_579812.1 | Immunoglobulin alpha Fc receptor isoform h |
|  |  | UP/SP | Hs | P24071.1 | Immunoglobulin alpha Fc receptor / IgA Fc receptor / CD_antigen=CD89 |
|  |  | NCBI | Oa | XP_004015480.2 | Immunoglobulin alpha Fc receptor isoform X1 |
|  |  | NCBI | Oa | XP_011950171.2 | Immunoglobulin alpha Fc receptor isoform X2 |
|  |  | NCBI | Oa | XM_027976143.1 | Fc fragment of IgA and IgM receptor (FCAMR), mRNA, predicted |
|  |  |  |  |  |  |
| IL-5 | IL5  Interleukin 5 | GB | Ch | ACH53209.1 | Interleukin 5 |
|  |  | GB | Oa | ACH53221.1 | Interleukin 5 |
|  |  |  |  |  |  |
| IL-5Rα | IL5R alpha  IL5 receptor alpha | NCBI | Ch | XP_017893977.1 | Interleukin-5 receptor subunit alpha isoform X2, predicted |
|  |  | NCBI | Oa | XP_004018366.2 | Interleukin-5 receptor subunit alpha isoform X1, predicted |
|  |  |  |  |  |  |
| CCR3 | CCR3  C-C chemokine receptor type 3 | NCBI | Ch | LOC5316646 | Capra hircus ARS1, ID: 10731 |
|  |  | ENA | Ch | JO419941.2 | Capra hircus Seql24210.Cahiliver mRNA sequence. |
|  |  | UP/SP | Oa | Q9N0M0 | CCR3 receptor |
|  |  | UP | Oa | W5PXW1 | G protein receptor F1_2 domain-containing protein |
|  |  |  |  |  |  |
| Eotaxin | Eotaxin  CCL11  C-C motif chemokine 11 | NCBI | Ch | XP_005693273.1 | Eotaxin |
|  |  | NCBI | Oa | XM_004012470.4 | Eotaxin (LOC101119832), mRNA, predicted |
|  |  |  |  |  |  |
| MBP | Major basic protein 1 / 2  PRG2  Proteoglycan 2  PRG3  Proteoglycan 3 | NCBI | Bt | NM_001098471.1 | Proteoglycan 3 (PRG3), mRNA. |
|  |  | NCBI | Ch | XM_018058942 | Proteoglycan 3-like (LOC102180520), mRNA, predicted |
|  |  | NCBI | Ch | XM_018058941.1 | Proteoglycan 3-like (LOC102180785), mRNA, predicted |
|  |  | NCBI | Hs | CR450311.1 | Full open reading frame cDNA clone RZPDo834F041D for gene PRG2, proteoglycan 2, bone marrow (natural killer cell activator, eosinophil granule major basic protein); complete cds; without stopcodon. |
|  |  | NCBI | Hs | NM_006093.4 | Proteoglycan 3, pro eosinophil major basic protein 2 (PRG3), mRNA. |
|  |  | NCBI | Oa | XM_027979081.1 | Proteoglycan 3-like (LOC101119933), mRNA, predicted |
|  |  | NCBI | Oa | XM_027979083.1 | Proteoglycan 3-like (LOC101105274), mRNA, predicted |
|  |  | NCBI | Oa | XM_027979084.1 | Proteoglycan 3-like (LOC101105022), mRNA, predicted |
|  |  |  |  |  |  |
| EPX | Eosinophil peroxidase  EPX | NCBI | Bt | XM_024980582.1 | Eosinophil peroxidase (LOC788751), mRNA, predicted |
|  |  | NCBI | Ch | XP_017919475.1 | Eosinophil peroxidase, low quality protein, predicted |
|  |  | NCBI | Ch | XM_018063986.1 | Eosinophil peroxidase (LOC102186992), mRNA, predicted |
|  |  | GB | Ch | LWLT01000022.1 | Capra hircus breed San Clemente chromosome 19, whole genome shotgun sequence. |
|  |  | NCBI | Hs | NM_000502.6 | Eosinophil peroxidase (EPX), mRNA. |
|  |  | GB | Oa | AMGL01017333.1 | Ovis aries breed Texel contig_17380, whole genome shotgun sequence. |
|  |  |  |  |  |  |
